# A remotely delivered, peer-led intervention to improve physical activity and quality of life in younger breast cancer survivors

**DOI:** 10.1007/s10865-022-00381-8

**Published:** 2022-12-07

**Authors:** Lauren S. Weiner, Stori Nagel, H. Irene Su, Samantha Hurst, Susan S. Levy, Elva M. Arredondo, Eric Hekler, Sheri J. Hartman

**Affiliations:** 1 Herbert Wertheim School of Public Health and Human Longevity Science, UC San Diego, La Jolla, CA USA; 2 UC San Diego Moores Cancer Center, 3855 Health Sciences Drive, La Jolla, CA USA; 3 Haus of Volta, Murrieta, CA USA; 4 Division of Reproductive Endocrinology and Infertility, Department of Obstetrics, Gynecology, and Reproductive Sciences, UC San Diego, La Jolla, CA USA; 5 School of Exercise & Nutritional Sciences, San Diego State University, San Diego, CA USA; 6 Institute for Behavioral and Community Health, San Diego State University Research Foundation, San Diego, CA USA; 7 School of Public Health, San Diego State University, San Diego, CA USA; 8 Center for Wireless & Population Health Systems, Qualcomm Institute, San Diego, CA USA

**Keywords:** Physical activity, Breast cancer, Exercise, Quality of life, Peer support, Mixed methods

## Abstract

Younger breast cancer survivors (YBCS) consistently report poorer quality of life (QOL) than older survivors. Increasing physical activity (PA) may improve QOL, but this has been understudied in YBCS. This single arm pilot study evaluated the feasibility and acceptability of a 3-month, peer-delivered, remote intervention to increase PA and improve QOL in YBCS. Data were collected from October 2019 – July 2020. Participants (n = 34, 43.1 ± 5.5 years old, 46 ± 34.4 months post-diagnosis, BMI = 30.2 ± 7.4 kg/m^2^) completed six video sessions with a trained peer mentor; self-monitored PA with a Fitbit activity tracker; and interacted with a private Fitbit Community for social support. At baseline, 3-and 6-months, participants completed QOL questionnaires and PA was measured through accelerometer (moderate-to-vigorous PA [MVPA]) and self-report (strength and flexibility). A parallel mixed-methods approach (qualitative interviews and quantitative satisfaction survey at 3-months) explored intervention feasibility and acceptability. One-way repeated-measures ANOVAs examined impacts on PA and QOL at 3-and 6-months. The intervention was feasible as evidenced by efficient recruitment, high retention, and adherence to intervention components. Remote delivery, working with a peer mentor, and using Fitbit tools were highly acceptable. From baseline to 3-months, participants increased time spent in objectively measured MVPA, strength, and flexibility exercises, and reported meaningful improvements to body image, fatigue, anxiety, and emotional support. A fully remote, peer-to-peer intervention is an acceptable and promising strategy to increase PA and improve QOL in YBCS. Refinements to the intervention and its delivery should be further assessed in future studies, toward the goal of disseminating an evidence-based, scalable intervention to the growing number of YBCS.

**Trial registration** Prospectively registered as NCT04064892.

**Supplementary Information:**

The online version contains supplementary material available at 10.1007/s10865-022-00381-8.

In women 20–49 years old, breast cancer is the most common cancer and the leading cause of cancer-related death (Howlader et al., 2019). Younger age is a risk factor for more advanced disease and aggressive treatment regimens that can impact fertility and lead to premature ovarian insufficiency and distressing menopausal symptoms (Rosenberg & Partridge, 2013). Younger survivors consistently report a higher impact of their cancer on quality of life (QOL) compared to older survivors (Champion et al., 2014). Younger women are often diagnosed during a life stage filled with building relationships, starting families and/or caring for aging parents, and establishing work-life balance (Rabin, 2017). Key psychosocial concerns include body image disturbances due to physical changes (e.g., hair loss, weight gain, surgical scars), sexual dysfunction, and fatigue (Howard-Anderson et al., 2012; Ruddy et al., 2013). To date, few evidence-based interventions have been identified to improve these aspects of QOL.

In older breast cancer survivors, physical activity (PA) can decrease fatigue and anxiety and may improve body image and sexual dysfunction (Mishra et al., 2012). Younger breast cancer survivors often reduce their PA during and after treatment (Howard-Anderson et al., 2012), and are less likely to be active than similar-aged women without cancer (National Cancer Institute, February 2019). For younger survivors, general barriers to PA, such as lack of time and competing demands, may be compounded by age-specific personal and professional stressors. This can include completing their education, establishing a career, and simultaneously caring for both young children and aging parents (Rabin, 2017). Given young survivors’ higher rates of depressive symptoms, fatigue, and persistent side effects from aggressive treatments (Rosenberg & Partridge, 2013), they may be more likely than older survivors to experience psychological barriers to PA (e.g., low motivation, emotional distress)(Rabin, 2017; Ventura et al., 2013). Taken together, these factors make it difficult for younger cancer survivors to start and maintain an exercise routine.

Few studies have tested PA interventions for young cancer survivors (Rabin et al., 2011; Rabin et al., 2016; Valle et al., 2013) and none have specifically focused on young breast cancer survivors. Only one trial used an objective measure of MVPA (Rabin et al., 2016), and none reported intervention effects on strength training or stretching & flexibility activities that are relevant to younger cancer survivors (Adams et al., 2021). None of these trials assessed intervention impacts on body image or sexual function. Further research is needed to determine feasible and acceptable strategies to promote PA and potential QOL benefits in younger breast cancer survivors.

This study leveraged a community based participatory research (CBPR) approach and emphasized equal partnership throughout the research process (Israel et al., 1998). The UC San Diego team collaborated with Haus of Volta, a non-profit founded by SN that promotes body positivity and well-being in younger cancer survivors. Details on the co-development of the research questions, study outcomes, and intervention in collaboration with Haus of Volta were previously published (Weiner et al., 2020). In brief, SN and the Haus of Volta Community Advisory Board served as primary resources in the design of the intervention and field-tested proposed intervention components. The UC San Diego and Haus of Volta teams collaboratively modified evidence-based intervention strategies to meet the needs of younger survivors. Modifications included 100% remote-delivery (rather than an initial in-person intervention session and in-person measurements) and adding the Pink Body Spirit Fitbit Community to facilitate social support. The primary aim of this study was to explore the feasibility and acceptability of the Pink Body Spirit PA intervention among younger breast cancer survivors. The secondary aim was to assess the preliminary impact of the intervention on PA (objectively measured MVPA and self-reported strength and flexibility) and QOL domains (body image, sexual function, fatigue, anxiety, depression, emotional support) that are highly prevalent and disruptive to younger survivors, but for which there are few evidence-based interventions.

## Methods

### Design & overview

This was a single arm feasibility trial of a 3-month, remotely delivered, peer-moderated PA intervention (Pink Body Spirit) among younger breast cancer survivors. Data were collected from October 2019 – July 2020. Protocol details were previously published (Weiner et al., 2020). Briefly, recruitment was led by Haus of Volta and focused on social media postings (Facebook and Instagram) in groups tailored to younger breast cancer survivors, including the Young Survival Coalition and Living Beyond Breast Cancer. Interested women contacted the study office and were phone screened for eligibility. Eligible participants were female breast cancer survivors who were diagnosed when aged 18–49 years old and currently 18–54 years old. Other inclusion criteria were: (1) completed surgery, chemotherapy, and/or radiotherapy at least 6 months prior to enrollment; (2) sedentary, defined as self-reporting < 60 min of MVPA per week; (3) accessible by phone or video chat; and (4) access to a Fitbit-compatible device with internet. Exclusion criteria were: (1) self-reported medical condition that could make it potentially unsafe to be in an unsupervised PA intervention; (2) currently pregnant; or (3) unable to commit to a 3-month intervention. The UC San Diego Institutional Review Board approved the study, and all participants provided written informed consent. After consent, participants were asked to complete baseline (T0) measures, consisting of online demographic and QOL questionnaires and wearing an ActiGraph accelerometer for 7 days. After finishing T0 measures, participants were mailed a Fitbit Charge 3 and completed a brief Zoom video call with research staff to set-up their Fitbit. Participants were then matched to a peer mentor based on mutual schedule availability and scheduled for their first mentoring session. Immediately after completing the 3-month intervention (3-months from baseline; T1) and 3 months after completing the intervention (6-months from baseline; T2) participants were again asked to wear the ActiGraph for 7 days and complete online questionnaires. At 3-months (T1), participants were individually interviewed to explore feasibility, acceptability, and satisfaction.

### 3-month physical activity intervention (Pink Body Spirit, months 1–3)

For a comprehensive intervention description, see Weiner et al. (Weiner et al., 2020). The Pink Body Spirit program was based on an effective exercise intervention for older survivors that was delivered in-person and via phone by a professional interventionist in a clinical research study (Hartman et al., 2018). The UC San Diego team and Haus of Volta partnered closely to adapt the evidence-based intervention to meet the needs of younger survivors (Weiner et al., 2020). The Pink Body Spirit program used peer mentoring, a Fitbit wearable activity tracker and companion app, and a private Fitbit Community to support behavior change. The intervention targeted constructs from Social Cognitive Theory (Bandura, 1986) and Control Theory (Carver & Scheier, 1982) associated with successful behavior change (Michie et al., 2009). Specific theroetical constructs and how intervention components target each construct are described in Table 1.

**Table 1:** Theoretical constructs targeted by the Pink Body Spirit physical activity intervention

Theoretical Construct	Peer Mentor Session Components	Fitbit Components
Self-monitoring^a,b^	• Orientation to self-monitoring & importance • Check-in about self-monitoring strategies and reinforce self-monitoring during each session	• Automatically tracks activity, option for manual input (swimming, etc.) • Graphical visualizations of daily, weekly, and monthly activity
Goal setting & review, action planning^a,b^	• Focus on incremental goals • Review progress toward goals • Goals & action plan updated as needed	• In-app goal setting for active minutes & steps, can be updated as goals change • Fitbit Exercise Calendar • Fitbit-recommended workouts
Comparison of performance to goals^b^	• Learn to compare current behavior with baseline behavior to detect small changes in behavior as they occur • Extra support from mentor based on Fitbit data compared to goal set	• Prompt review of behavioral goals • Rich visualizations of behavior over time relative to defined goals • Fitbit Challenges
Opportunities & Barriers, Problem solving^a^	• Identify barriers to physical activity • Generate strategies to overcome barriers and increase facilitators • Intervention toolbox	• Fitbit community board postings from mentors and other participants
Outcome expectations^a^	• Peer mentor provides information about positive outcomes of increasing activity, e.g., reducing risk of recurrence & improving quality of life	• Fitbit app provides information about benefits of exercise
Self-efficacy^a^	• Identify previous success sticking to a goal (previous mastery) • Encouraged to feel proud of their ability to achieve & maintain goals (verbal persuasion) • Tips to stay motivated • Incremental goals	• Reminders of previous successes in app • Motivational messages in app and on tracker
Social support^a^	• Matched with same peer mentor for entire 12-week intervention • Identifying helpful and unhelpful sources of social support for behavior change (e.g., family, friends, healthcare team)	• Support from other participants and peer mentors in Fitbit Community Group • Fitbit Leaderboard & Challenges
Rewards/Recognition/Reinforcement^a^	• Verbal and written praise for meeting goal • Create a plan to reward self for achieving goal	• In-app badges, banners, vibrations (on tracker) and emails from Fitbit for meeting goals • Likes, comments, and mentions in Fitbit Community Group • Fitbit Leaderboard & Challenges
Feedback on performance^a,b^	• Feedback from peer mentor about behavior using activity data from Fitabase	• In-app feedback • Feedback on Fitbit tracker • Weekly progress emails from Fitbit

*Note.*^a^ Theoretical construct is part of Social Cognitive Theory; ^b^ Theoretical construct is part of Control Theory

The 3-month, technology-supported program was fully remote to help address common barriers to exercise among younger cancer survivors related to scheduling, lack of time, and large geographical distances between young survivors (Rabin, 2017). A key consideration for utilizing peer mentors was Haus of Volta’s interest in learning how younger survivors can help each other increase their activity and improve their health. Peer mentors are trained individuals who have shared experiences to provide knowledge, emotional, social, and or/practical help to support others. In older cancer (Pinto et al., 2015) and chronic disease populations, peer led-programs have been effective for promoting and maintaining PA (Buman et al., 2011; Clark et al., 2012), and using peers to deliver behavioral interventions may extend the reach of evidence-based interventions into the community (Rini et al., 2018). The use of peer mentors and a private Fitbit Community were also intended to meet younger survivors’ preferences for behavioral interventions that provide peer support (Rabin et al., 2013).

For this project, five younger breast cancer survivors (diagnosed < 50 years old and currently < 55 years old, completed surgery, chemotherapy, and/or radiation) were trained by LSW and SJH to deliver the program to fellow younger survivors as peer mentors. Peer mentors were selected by Haus of Volta and paid for their time by the research project grant. Peer mentors were trained in research ethics, privacy, and data safety; exercising safely and adverse events; motivational interviewing; and intervention protocol delivery (Weiner et al., 2020). All trainings were conducted via Zoom video calls, except for one component of the motivational interviewing training that had a virtual or in-person option.

Participants completed six Zoom video calls with their trained peer mentor over the 3-month intervention. Topics for the initial 45-minute session included basic introductions; self-monitoring with the provided Fitbit Charge 3; how to use the in-app, private Fitbit Community; and exercise goal setting. Introductions were structured to keep the focus on the mentee and her behavior change plan. Peer mentors used motivational interviewing techniques to help each participant create a personalized exercise goal and action plan. Motivational interviewing is a collaborative, goal-oriented method of communication designed to strengthen an individual’s motivation for and movement toward a specific goal by eliciting and exploring the person’s own arguments for change (Martins & McNeil, 2009). Motivational interviewing approaches have been shown to positively support physical activity improvements for people with chronic conditions (O’Halloran et al., 2014), particularly when combined with wearable activity trackers (Pudkasam et al., 2021). The overall goal for all participants was to meet the guidelines for cancer survivors of at least 150 minutes of MVPA per week by the end of the 3-month intervention period (Campbell et al., 2019). Participants were also supported in increasing strength training and stretching and flexibility exercises. Peer mentors utilized motivational interviewing approaches to support personalized goal setting and encouraged gradual PA increases over time to prevent injuries.

A total of 5 follow-up video sessions (20–30 min each) were scheduled every other week over the 3-month program to review progress and update the personalized exercise goal and/or action plan as needed. Participants were encouraged to wear the Fitbit as often as possible, but at minimum, to wear it while they were exercising, so that their mentor would be able to see their Fitbit data and provide feedback throughout the intervention. Peer mentors accessed Fitbit data via Fitabase (Small Steps Labs, LLC), a web-based software that collects activity and heart rate data from the Fitbit cloud. Peer mentors used Fitbit activity data to support performance feedback and goal review during biweekly follow-up sessions, and to identify participants in need of additional support between scheduled sessions.

To further enhance social support, participants were asked to create a new post in the in-app, private Fitbit Community at least once per week. Participants worked with the same peer mentor for the entire program to encourage rapport and continuity. To maximize flexibility and convenience, mentors and mentees could communicate through multiple channels, including text, call, email, and private Fitbit messages.

### Postintervention follow-up period (months 4 to 6)

The three-month postintervention follow-up period (months 4–6) explored engagement with the program beyond the 3-month intervention. At the end of the intervention, peer mentors strongly encouraged participants to continue using their Fitbit to track their activity and continue participating in the Fitbit Community. However, there were no scheduled contacts or video sessions, nor did peer mentors monitor Fitbit activity during the follow-up period.

### Measures and outcomes

#### Feasibility and acceptability

##### Recruitment and retention

We tracked the number of women who were phone screened, referral source for each, and number of eligible women who enrolled, to determine if we met our feasibility metric of enrolling ≥50% of screened individuals within the 5-month recruitment time frame and retaining ≥80% of those enrolled through 6-month measures.

##### Adherence

Adherent was defined as meeting two of three of the following metrics: (1) wearing the Fitbit on > 75% of days, (2) completing > 75% of intervention sessions, or (3) posting in the Fitbit Community at least once per week on > 75% of weeks. Intervention session completion was tracked by peer mentors in a study database. Adherence to posting in the Fitbit Community was measured through manual review of posts. Fitbit data from the Charge 3 were used to calculate daily adherence to wearing the Fitbit. Fitbit data were wirelessly uploaded to the user’s personal Fitbit account and downloaded for analysis through Fitabase. Non-wear time was determined based on lack of heart rate or activity (steps or intensity) at any given minute (Nelson et al., 2019). Consistent with previous research (Hartman et al., 2018), we a priori defined daily adherence to wearing the Fitbit as ≥600 min (10 h) of heart rate data or ≥1 min of “Fairly Active Minutes” or “Very Active Minutes” (MVPA). This definition for an adherent Fitbit wear day was selected because the intervention emphasized using the Fitbit specifically to self-monitor intentional exercise; therefore, wearing the Fitbit only to measure their PA, even if it was not worn all day, was considered adherent per intervention instructions.

##### Mixed methods evaluation

A parallel mixed methods approach (QUAL + QUAN) explored feasibility, acceptability, and satisfaction of the intervention from participants’ perspectives. Qualitative and quantitative data were separately collected, analyzed, and reported. Complementarity of the two sets of findings were explored in the interpretation (Greene et al., 1989). A parallel approach was selected because qualitative and quantitative data collection were equally important to achieve the study aims, and each type of data had the potential to yield unique information about the intervention experience. Additionally, using mixed methods facilitated greater assurance about the validity of the research questions and the amount of data collected to answer these questions.

##### Post-intervention qualitative interviews

At T1 (3-months), individual, semi-structured interviews explored overall perceptions of the program and specific components (e.g., peer mentors, Fitbit, Fitbit Community), perceived benefits, the remote delivery format, and suggestions for improvement (see Supplementary Material 1). Each 30 to 45 min interview was conducted by LSW via Zoom video call. Interviews were recorded and transcribed verbatim using InqScribe transcription.

#### Change in physical activity

Change in minutes per week of MVPA was measured with the ActiGraph GT3X+ (ActiGraph, LLC), a hip-worn accelerometer that captures movement and intensity of activity and has been validated against heart rate telemetry and total energy expenditure (Melanson & Freedson, 1995; Plasqui & Westerterp, 2007). The GT3X + provided second-level estimates of activity that were categorized into minutes spent in sedentary, light, moderate, and vigorous activity using Freedson cut-points (Freedson et al., 1998). Sufficient wear time was defined as five days with ≥ 600 min of wear time or 3000 min (50 h) across four days. Change in days per week of strength training and stretching & flexibility exercises were measured using two items adapted from the Exercise Vital Sign (Coleman et al., 2012; Quiles et al., 2019). Participants self-reported the number of days in the past week they did each type of activity: (1) muscle strengthening exercises (e.g., weight lifting, bodyweight exercises or resistance training; hereafter referred to as “strength training”) and (2) stretching and flexibility exercises.

#### Change in quality of life

**Body image** was measured with the 10-item Body Image Scale (BIS). Total scores range from 0 to 30; higher scores indicate poorer body image (Hopwood, 2001). Total scores ≥ 10 have been considered indicative of body image distress (Hopwood, 2001), but minimal clinically importance differences over time have not been established. The BIS has shown high reliability, clinical validity, and sensitivity to change in older breast cancer survivors (Lewis-Smith et al., 2018). The BIS showed high internal consistency in this study (Cronbach’s α = 0.93 at T0, 0.93 at T1, and 0.92 at T2).

**Sexual function** was measured with the 19-item Female Sexual Function Index (FSFI). Total scores range from 2 to 36; higher scores indicate better sexual function over the past 4 weeks (Rosen, 2000). A total score ≤ 26 has been validated as a cutoff for clinically-relevant dysfunction (Wiegel et al., 2005). The FSFI has shown good reliability and validity in sexually active breast cancer survivors (Bartula & Sherman, 2015), and demonstrated high internal consistency in the current study (Cronbach’s α = 0.98 at T0, T1, and T2).

**PROMIS measures**. Fatigue, anxiety, and depression were assessed via computer-adaptive testing versions of the PROMIS Cancer v1.0 measures for each construct (Jensen et al., 2017). Emotional support was assessed through the PROMIS v2.0 measure (Hahn et al., 2014). Each PROMIS measure yields a standardized t-score with a mean of 50 and a standard deviation of 10. Higher scores indicate higher levels of the construct. The fatigue, anxiety, and depression scales have shown responsiveness to change over time in prospective (Jensen et al., 2017) and intervention (Hartman et al., 2019) studies in cancer survivors.

### Analyses

#### Feasibility and acceptability

##### Adherence

Descriptive statistics and frequencies (mean [SD] and n [%] where applicable) were used to calculate the three adherence metrics (proportion of days the Fitbit was worn, proportion of intervention sessions completed, and proportion of weeks a Fitbit Community post was created).

##### Post-intervention qualitative interviews (T1)

Rapid qualitative analysis techniques, including matrix displays and triangulation, were used to analyze semi-structured interviews (Averill, 2002; Beebe, 2005). A multi-step, iterative approach allowed us to feed insights back into the ongoing study to improve it (Hamilton, 2013). See Supplementary Material 1 for full description of the rapid qualitative analysis approach.

##### Post-intervention quantitative satisfaction questionnaires (T1)

Descriptive statistics and frequencies were used to analyze responses to close-ended items. Responses to open-ended items were reviewed by LSW and SJH to identify themes. Once team consensus was reached, findings from open- and close-ended items were synthesized and summarized narratively by category (peer mentor, Fitbit tracker, Fitbit Community, overall impressions).

##### Mixed methods interpretation

Qualitative interviews and quantitative surveys were analyzed separately. The two sets of results (QUAL + QUAN) were triangulated by area of feedback (e.g., feedback on peer mentor, Fitbit & Fitbit Community, perceived benefits) to examine how each set of data corroborated and strengthened ideas in the other set and to explore complementarity of the findings. The QUAL + QUAN results were then synthesized for interpretation. The mixed methods synthesis of the QUAL + QUAN results is presented in the results section of this manuscript.

Moreover, the collective co-interpretation of results is a critical aspect of the analytic approach in CBPR (Israel et al., 1998). Thus, LSW led a structured discussion with Haus of Volta and the peer mentors to elicit their reactions to and interpretations of the findings related to feasibility and acceptability of the pilot intervention and changes in PA and QOL. The interpretations presented in the Discussion section of this manuscript reflect the collective co-interpretation of results by all partners.

#### Change in physical activity and quality of life

Data were analyzed using R statistical software (version 4.0.2) (R Core Development Team, 2017). Descriptive statistics and frequencies were calculated for each outcome of interest at each time point. For self-reported outcomes, values greater than ± 2.58 SDs from the mean (< 99th percentile or < 1st percentile) were considered outliers and were excluded from all analyses (Weiner et al., 2012). Separate one-way repeated measures ANOVAs assessed change in each outcome of interest over time. An alpha level of 0.05 was used for statistical significance. For significant ANOVAs, Tukey post hoc tests were used to determine differences between time points. Eta-squared effect sizes were also calculated. Correlations between key outcome variables and potential covariates (BMI, age, and time since diagnosis) were explored. All variables were weakly correlated (r = 0.01 to r = 0.36). Therefore, none of the potential covariates were included in the analyses.

##### Missing data

An intention to treat approach was used to avoid overestimation of intervention effects on PA and QOL. The “last observation carried forward” approach was used. For the three participants with missing data at T1 and T2, the value from the last completed assessment (baseline) was carried forward or imputed. Where self-reported data were incomplete (i.e., one or more survey items were missing from a scale), the mean of the other items in the scale (or subscale) was imputed.

## Results

### Feasibility

#### Recruitment & retention

**Fig. 1:**
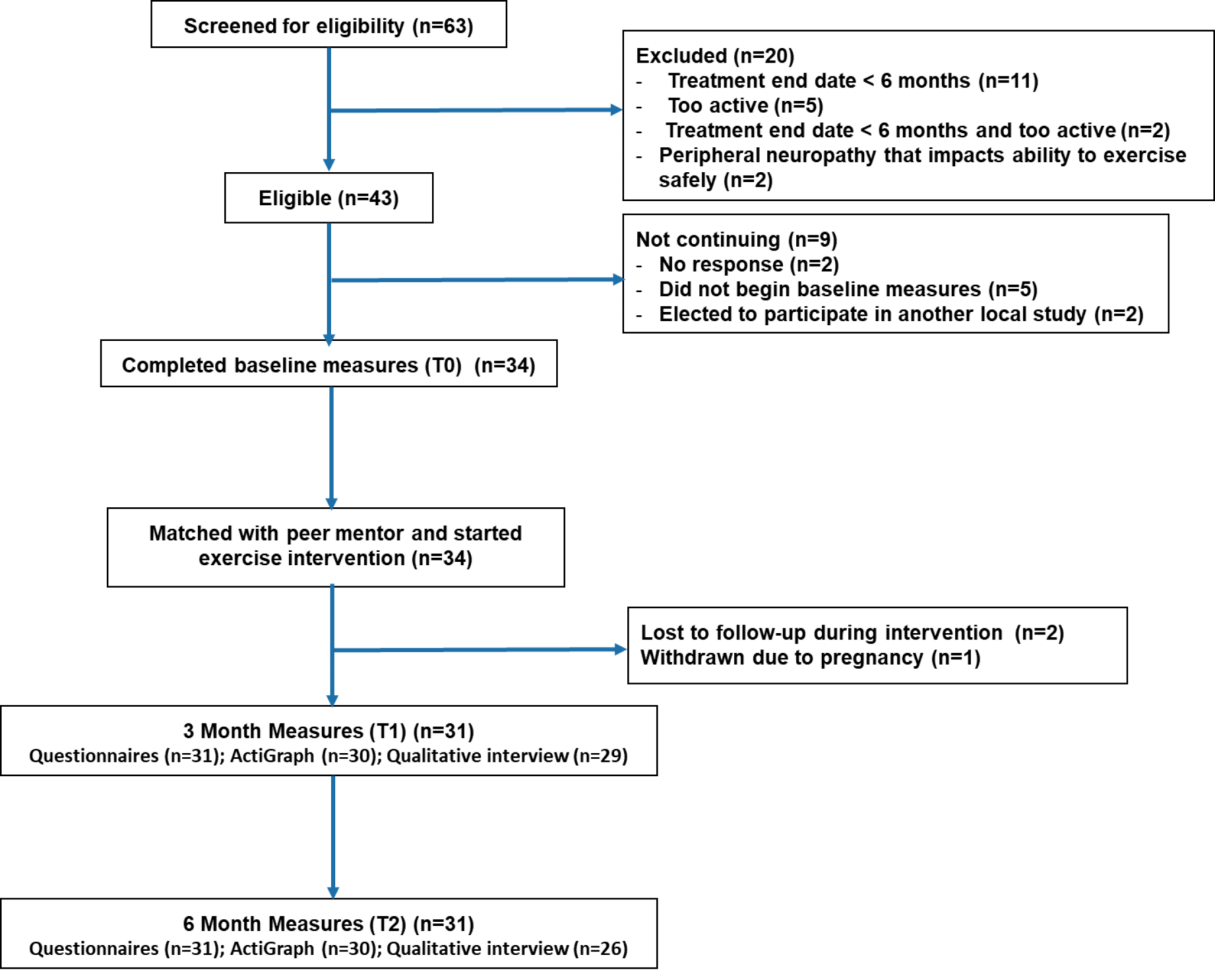
CONSORT diagram showing participant recruitment and retention throughout the trial

Recruitment was completed over 3 months (September to December 2019). Of the 63 women screened, 43 (68%) were eligible. The most common reason for ineligibility was treatment end date within the past 6 months (n = 13). Of the 43 eligible women, 34 were enrolled, defined as completing T0 measures and at least one intervention session. Thirty-one women were retained for T1 measures. All 31 women who completed T1 measures also completed T2 measures, resulting in a 91% retention rate (See CONSORT, Fig. 1). Most women (76%) were recruited through social media, followed by support group referral (12%), referral from another participant (9%), and UC San Diego Cancer Center Patient Navigator referral (3%).

Table 2 shows demographic and clinical characteristics of the sample. Women were, on average, 43.1 ± 5.5 years old, predominantly white (88%), non-Hispanic (85%), with an average BMI of 30.2 ± 7.4 kg/m^2^. On average, participants were 46 ± 34.4 months post-diagnosis. At baseline (T0), women engaged in an average of 90.2 ± 49.9 min of accelerometer measured MVPA per week, five participants (15%) self-reported strength training exercises at least one day per week, and seven (21%) self-reported stretching or flexibility exercises at least one day per week. Participants comprised a nationwide sample representing 15 different states across four time zones.

**Table 2:** Baseline (T0) self-reported demographic and cancer characteristics (N = 34)

Demographic characteristic	Value
Age (years), mean (SD), range	43.1 (5.5), 33.6–51.8
Body Mass Index (kg/m^2^), mean (SD), range	30.2 (7.4), 21.0–45.0
Race^a^, n (%)	
White	30 (88.2)
Black or African American	4 (11.8)
American Indian or Alaska Native	1 (2.9)
Ethnicity, n (%)	
Hispanic	5 (14.7)
Non-Hispanic	29 (85.3)
Education, n (%)	
High school or GED	2 (5.9)
Some college or Associate’s degree	13 (38.2)
College or Graduate degree	19 (55.9)
Marital status, n (%)	
Never Married	5 (14.7)
Divorced or separated	4 (11.8)
Presently married or living with partner	25 (73.5)
Living situation, n (%)	
Live alone	2 (5.9)
Live with spouse or partner	4 (11.8)
Live with spouse or partner and children	20 (58.8)
Live with children (no spouse or partner)	4 (11.8)
Live with parents or other relatives	4 (11.8)
Employment^b^, n (%)	
Employed full-time	21 (61.8)
Employed part-time	5 (14.7)
Homemaker, raising children, caring for others	7 (20.6)
Student	2 (5.9)
Volunteer	2 (5.9)
Not working	3 (8.8)
**Cancer characteristic**	
Time since diagnosis (months), mean (SD), range	46.0 (34.4); 9–160
Disease stage, n (%)	
Stage I	8 (23.5)
Stage II	15 (44.1)
Stage III	10 (29.4)
Stage IV	1 (2.9)
Chemotherapy, n (%)	24 (70.6)
Radiation, n (%)	18 (52.9)
Breast cancer surgery, n (%)	
Lumpectomy	9 (26.5)
One-sided mastectomy	5 (14.7)
Bilateral mastectomy	20 (58.8)
Hormonal therapy, n (%)	25 (73.5)
Currently taking hormonal therapy	17 (68.0)
Not currently taking, currently prescribed	4 (16.0)
Not currently taking, completed	4 (16.0)
HER2 + infusions (e.g., Herceptin), n (%) ^c^	9 (26.5)
Currently receiving infusions	1 (2.9)
Not currently receiving infusions	8 (23.5)
Lymphedema, n (%)	
Ever diagnosed with lymphedema	13 (38.2)
Currently experiencing lymphedema	7 (20.6)
Hysterectomy, n (%)	6 (17.6)
Oophorectomy, n (%)	11 (32.4)

*Note.* SD = Standard Deviation; ^a^ Total exceeds 100% because participants could self-identify as multiple races; ^b^ Total exceeds 100% because participants could self-report more than one type of employment; ^c^ HER2 + = human epidermal growth factor receptor 2

#### Adherence

Adherence to intervention components is reported in Table 3. Most women (85.3%) wore the Fitbit on at least 75% of days; 79.4% of participants completed at least 75% of peer mentor sessions; and 5.9% initiated a new post in the Fitbit Community on at least 75% of intervention weeks. Almost a third (29%) of women initiated a new post on at least 50% of intervention weeks. Overall, 76.5% of participants met at least two of the three adherence metrics and were considered adherent.

**Table 3:** Adherence to intervention components among Pink Body Spirit participants (N = 34)

Intervention component	Adherence definition	Proportion of participants who were adherent
Fitbit	≥600 min (10 h) of heart rate data or logging ≥1 min of “Fairly Active Minutes” or “Very Active Minutes” (MVPA) on at least 75% of days	85.3%
Intervention sessions	Completing at least 75% of the 6 planned peer mentoring sessions	79.4%
Fitbit Community	Initiating at least one new post on at least 75% of weeks	5.9%
Overall	Meeting the adherence criteria for at least two of the three intervention components above	76.5%

### Acceptability – mixed methods synthesis of QUAL interviews + QUAN satisfaction questionnaires

#### Peer mentoring

Most participants reported positive experiences working with a peer mentor who had “been in the same shoes” and could “understand the different levels of frustration…when you’re coming out of treatment.” One woman shared: “At least for me, survivorship has been in some ways harder than active treatment. I think a lot of it is just mentally wanting to get back to where you were before…but having the peer mentor was great, because it was relatable, and she had a really good attitude as far as being able to motivate, but also having empathy.” Many participants sought deeper relationships with their mentors. One woman elaborated: “When I was talking with my mentor, I feel like it would have been nice to have known her story… just so we can relate a little bit more… There aren’t many young people that you come in contact with that have breast cancer, so to actually be chatting with somebody, just to have that connection.”

Participants appreciated the convenience of scheduling virtual mentoring sessions and felt that video calls helped them feel more connected than talking on the phone, because they could make eye contact and see facial expressions. Many participants felt accountable to their mentors; knowing their mentor could see their Fitbit activity increased their motivation to stay on-track. Most women felt the number and frequency of mentoring sessions worked well; some wanted to meet more often, while others sensed they wouldn’t have time for additional sessions. Women also had varying preferences for communication with their mentor (e.g., Fitbit message, text, email). Participants valued clear and consistent preparation, communication, and follow-through from their mentors, whom many described as “professional,” “prepared”, and “organized”. Yet not all women felt this way. A few were “frustrated” or “irritated” by logistical or scheduling challenges, such as when their mentor missed or was late to a session. Participants who reported frequent communication with their mentor between sessions liked it; those who said they didn’t receive much contact between sessions wanted more interaction.

#### **Fitbit & Fitbit Community**

Women generally found it helpful to track active minutes, though a few preferred tracking steps. While participants did not initiate posts in the Fitbit Community often, they engaged in other ways that facilitated social support and other behavior change techniques, including reading others’ posts, viewing the Leaderboard, and joining Fitbit Challenges. Women described the Fitbit Community as “motivating”, “non-competitive” and a “place to be held accountable.” Many shared a desire to forge deeper connections with the other participants and mentors in the study and felt that more activity and engagement in the Fitbit Community would have fostered this. The qualitative interviews also revealed numerous barriers to posting in the Fitbit Community, including discomfort posting personal information to an unknown audience, insufficient time to post, forgetting to post, or difficulty accessing the Community within the Fitbit app.

### Changes in physical activity

Participants significantly increased minutes per week of objectively measured MVPA (F (2, 58) = 5.94, *p* < 0.005, η^2^ = 0.06), days per week of self-reported strength training (F (2, 62) = 11.20, *p* < 0.0001, η^2^ = 0.14) and days per week of self-reported stretching and flexibility exercises (F (2, 60) = 8.46, *p* < 0.0001, η^2^ = 0.11) over time (see Table 4 for full results). Participants increased their MVPA, on average + 39.7 min/week (95% CI: -6.8 to + 86.2) from T0 to T1, and minutes per week of MVPA remained stable from T1 to T2. Similarly, strength training and stretching & flexibility exercises each increased by + 0.8 days per week from T0 to T1, and days per week spent in both activities remained stable from T1 to T2.

**Table 4:** Physical activity and quality of life outcomes at T0, T1, and T2 and changes between time points among Pink Body Spirit participants

		Time point			Change			Repeated Measures ANOVA		
		T0	T1	T2	T0 to T1	T0 to T2	T1 to T2	F value	p-value	n^2^
Outcome	N	M (SD)	M (SD)	M (SD)	M (SD)	M (SD)	M (SD)			
MVPA^a^	30	90.2 (49.9)	129.9 (84.2)	127.4 (86.8)	+ 39.7 (63.4)	+ 37.2 (76.4)	-2.5 (71.6)	5.94	< 0.005	0.06
Strength training^b^	32	0.1 (0.3)	0.9* (1.3)	1.1* (1.4)	+ 0.8 (1.2)	+ 1.0 (1.4)	+ 0.2 (1.2)	11.2	< 0.001	0.14
Stretching & flexibility^c^	31	0.23 (0.5)	1.06* (1.6)	1.32* (1.7)	+ 0.8 (1.2)	+ 1.1 (1.5)	+ 0.3 (1.7)	8.46	< 0.001	0.11
Body image^d^	34	17.2 (8.4)	13.2 (8.2)	11.3* (7.1)	-4.0 (5.7)	-5.9 (6.1)	-1.9 (4.1)	21.24	< 0.001	0.09
Sexual function^e,f^	34	14.8 (10.7)	15.2 (11.0)	15.2 (11.9)	+ 0.4 (6.2)	+ 0.1 (5.9)	-0.1 (7.0)	0.117	< 0.889	0.004
Fatigue^g,h^	33	60.2 (8.2)	54.3* (8.1)	54.0* (9.0)	-5.8 (8.5)	-6.1 (8.4)	-0.3 (7.5)	11.94	< 0.001	0.1
Anxiety^g,i^	33	60.7 (7.6)	57.1 (9.5)	57.0 (9.3)	-3.6 (9.7)	-3.7 (7.5)	-0.1 (9.8)	3.58	< 0.034	0.04
Depression^g^	34	54.0 (9.0)	52.2 (9.2)	51.7 (8.0)	-1.8 (7.1)	-2.0 (6.2)	-0.5 (6.6)	2.18	< 0.121	0.01
Emotional support^g^	34	46.5 (8.2)	49.2 (8.6)	50.8 (9.6)	+ 2.7 (6.4)	+ 4.3 (6.3)	+ 1.6 (6.4)	7.86	< 0.001	0.04

*Note.* M = Mean; SD = Standard Deviation

^a^ MVPA = Moderate to Vigorous Physical Activity measured with ActiGraph accelerometer, min/week, removed 4 outliers due to high MVPA at T0; ^b^ days/week, removed 2 outliers with high strength training at T0; ^c^ days/week, removed 3 outliers with high stretching & flexibility at T0 or T1; ^d^ Score range = 10–40; ^e^ score range = 2–36; ^f^ Removed 12 participants who were not sexually active at T0; ^g^ PROMIS measures reported as standardized t-score with mean = 50 and SD = 10; ^h^ Removed 1 outliers with low fatigue at T0; ^i^ Removed 1 outlier with low anxiety at T0; *significantly (p < 0.05) different from T0; η^2^ = eta-squared effect size

### Changes in quality of life

#### Quality of life – quantitative findings

At T0, participants reported high levels of body image distress, sexual dysfunction, fatigue, and anxiety. Participants reported improvements in body image (F (1.69, 55.79) = 21.24, *p* < 0.001, η^2^ = 0.09), fatigue (F (2, 64) = 11.94, *p* < 0.0001, η^2^ = 0.10), and anxiety (F (2, 64) = 3.58, *p* < 0.034, η^2^ = 0.04) over time. Participants also reported increases in emotional support over time (F (2, 66) = 7.91, p < 0.001, η^2^ = 0.04). Participants reported reduced anxiety and fatigue at T1 that remained stable at T2. Participants reported fewer body image concerns and higher emotional support at T1 and continued to show improvement at T2. There were no significant changes over time in depression (F (2, 66) = 2.18, *p* < 0.121, η^2^ = 0.01) or sexual function (F (2, 66) = 0.117, *p* > 0.889, η^2^ = 0.004). (Table 4).

#### Quality of life – qualitative findings

Participants described many physical health benefits such as improved stamina, feeling physically stronger, and less fatigue. Benefits to mental health included positivity and vitality, improved mood, and lower stress. Some women also described a more positive self-image, self-confidence, and a greater desire to improve their overall health. Multiple participants described that participating in this study was among their first experiences engaging in self-care since their diagnosis. One shared: “It made me want to look better. I kind of threw that out the window for a long time because I’ve had so much surgery and stuff…this study has given me a pathway to start to rebuild myself and my life, and that’s huge, that’s really huge.”

## Discussion

This study explored the feasibility and acceptability of a fully remote PA intervention in younger breast cancer survivors. Results showed it was feasible to recruit and retain younger breast cancer survivors into the 3-month intervention. The technology-based, peer-led approach was acceptable, as demonstrated by high adherence to intervention components and positive participant feedback. Women increased objectively measured and self-reported PA and experienced meaningful improvements to QOL domains that are commonly impaired in younger survivors, including body image, fatigue, anxiety, and emotional support.

### Recruitment and retention

Given the focus on scalability and to increase ecological validity, there were few eligibility criteria. The high proportion of women eligible in a short time frame suggests that the recruitment methods yielded suitable women very efficiently. Our success with social media recruitment aligns with other studies (Gorman et al., 2014; Stark et al., 2019) and highlights the importance of community members helping to define *and* implement recruitment strategies.

### Adherence, engagement, and acceptability

Overall, participants were highly adherent to completing mentoring sessions and reported positive experiences working with their mentors. Participants noted that working with a peer who was diagnosed at a similar age and life stage was unique from other survivorship programming; however, many sought deeper connections with their mentors. Peer mentors’ training was focused on delivery of the exercise intervention and introductions were structured to keep the focus on the mentee and her behavior change journey. Future iterations of this program should train mentors in how to appropriately share their own experiential knowledge about exercising after breast cancer treatment to leverage the mutual identification only a peer mentor can provide (Ginis et al., 2013; Snyder & Pearse, 2010).

Although women did not frequently initiate posts in the Fitbit Community, and thus were not considered adherent to completing this intervention component per our a priori definition, many participated in other ways that facilitated social support and behavior change techniques associated with success in a PA intervention (Finne et al., 2018; Grimmett et al., 2019). For instance, women read others’ posts to gain ideas for PA and how to overcome barriers, viewed the Leaderboard, and joined Fitbit Challenges to receive rewards and recognition. Many participants felt the Community was motivating, inspiring, and an additional source of accountability. While virtual communities have facilitated social support in the context of behavioral interventions among people with breast cancer and other chronic conditions (Hossain et al., 2021), our sample may have been too small to reach the “critical mass” of participants needed to generate and sustain an active and vibrant virtual community (McKay et al., 2001). Taken together, these data suggest that within the context of our study, participants highly valued the social connections built through peer mentoring, and one-on-one mentoring was more acceptable than the Fitbit Community in supporting these connections. However, given the small sample size, these results may not be generalizable beyond the present study population.

While most participants stated that the fully remote format was more convenient than in-person, many women faced numerous conflicting priorities and schedule-related barriers that hindered their engagement. The next iteration of this intervention could consider more asynchronous peer mentoring contacts (e.g., email, text, Fitbit private message, etc.) that may more easily fit into busy schedules and require less coordination than video calls. Participants had high adherence to wearing the Fitbit (> 85% wore it on at least 75% of intervention days), and described that sharing their Fitbit activity data with their mentor helped them feel motivated and accountable. While data were collected in real time, this intervention did not provide real-time feedback. To meet participants’ preferences and increase scalability, future studies could consider using technology to deliver tailored, data-driven feedback. The frequency and intensity of intervention contacts to promote accountability could be adaptive and tailored to participants’ preferences, or adjusted dynamically based on a participant’s progress toward their individual PA goals, such as through “continuous tuning interventions” that use a participant’s data and real-time optimization algorithms to “tune” the intervention to meet their individual needs (Chevance et al., 2021; Hekler et al., 2020). Using algorithms and technology to facilitate feedback loops could also increase the likelihood that intervention components are delivered consistently and with higher fidelity (Moller et al., 2017). Related concepts are currently being tested in an ongoing study in young cancer survivors using activity tracker data to determine the frequency and content of text messages and to tailor feedback to participants (Valle et al., 2021). Finding ways to support behavior change in light of many competing demands remains crucial for successfully intervening in younger breast cancer survivors.

### Preliminary effects on physical activity and quality of life

While women significantly increased MVPA, strength training, and stretching & flexibility exercises from baseline to 3-months, most participants’ activity levels remained below the target of 150 min per week, which was based on PA guidelines to improve cancer-related health outcomes (Campbell et al., 2019). Effect sizes for PA outcomes were quite small, which is not unexpected for this exploratory, one-arm study. Of the three published studies in younger cancer survivors (Rabin et al., 2011; Rabin et al., 2016; Valle et al., 2013), only one trial found significant between-group differences in change in PA between the intervention and control groups (Rabin et al., 2016), and there was variable engagement with intervention components. To our knowledge, this is the first trial in younger breast cancer survivors to report increases in objectively measured MVPA. Future studies should assess if a longer intervention period may support greater increases to meet PA guidelines. Additionally, a limitation of accelerometers is that they do not capture context of PA; therefore, it was not possible to discern how much of the observed increase in PA was intentional versus unintentional activity. Future research could consider using measures that capture context surrounding the PA to support the development of improved interventions and inform our understanding of young breast cancer survivors’ tolerance for exerting energy for movement, above and beyond intentional PA.

Participants also reported meaningful improvements in several QOL domains. Specifically, participants achieved clinically meaningful improvements (Yost et al., 2011) in fatigue and anxiety, measured with the PROMIS-Cancer measures, that were consistent with benefits described in the qualitative interviews. Participants also reported improved self-image and body confidence during qualitative interviews that were reflected on the BIS. Our sample was highly distressed at baseline, and despite reductions in anxiety, fatigue, and body image over time, participants continued to experience high levels of distress at 6 months. Given the burden of QOL impairments evidenced in this study and in the broader literature (Paterson et al., 2016), as well as the lack of evidence-based interventions, more research is needed in this area.

Additionally, participants reported increases in emotional support, complementing the mixed methods findings that women were accepting of intervention components targeting social support, particularly peer mentoring. Among younger cancer survivors, who often report social isolation, social support can be a facilitator of health behavior change (Pugh et al., 2019). This study was not designed to understand the impact of individual intervention components, such as features that provide social support (e.g., Fitbit Community, peer mentoring) on change in PA or QOL. Future research should consider using alternative experimental approaches, such as the Multiphase Optimization Strategy (MOST), to systematically determine which individual components are having the intended effect in an intervention and which doses of each component lead to the best outcomes (Collins et al., 2007). Finally, congruence between subjective QOL improvements reported in qualitative feedback and improvements on validated quantitative measures corroborates the meaningfulness of these benefits in the context of participants’ lives and enhances the validity of the findings.

The study sample showed high levels of sexual dysfunction at all time points, and there were no observed changes to sexual function over time. However, over one-third of participants reported no sexual activity at baseline. There are known threats to validity when using the FSFI to measure sexual function in women without recent sexual activity (Boehmer et al., 2012; Meston et al., 2020). Further studies using larger samples and measures less dependent on current sexual activity should assess if PA can improve this highly prevalent concern in younger breast cancer survivors.

## Limitations

This study occurred during the COVID-19 pandemic. All participants completed baseline measures and about two-thirds of participants completed 3-month measures prior to the start of the pandemic (defined as March 12, 2020). All participants completed 6-month measures during the pandemic. Since measures were administered remotely, we were able to collect all outcome data as planned. However, external factors related to the pandemic may have differentially impacted adherence, engagement, and delivery of the intervention. Some peer mentors experienced changes to their jobs that resulted in more flexibility or availability for peer mentoring, while others had to balance increased familial responsibilities or other life circumstances that limited their capacity. The nationwide participant sample drew from 15 geographically diverse states with different COVID-19 restrictions. There is evidence that the pandemic and associated restrictions had detrimental impacts on self-reported and objectively assessed PA compared to pre-COVID-19 activity levels among the general population (Stockwell et al., 2021) and in cancer patients, though some people also increased their PA (Himbert et al., 2021). Participants reported many changes to their lives in line with documented barriers to PA during the COVID-19 pandemic (Roche et al., 2022), including changes to the hours, location, amount of work, or employment status; changes to the time and/or location of their schooling or that of their family members; changes to their family life and caregiving responsibilities; and changes to their relationships. Some women reported having more time to exercise and others had less time and/or reduced access to exercise facilities. Reduced PA during COVID-19 has been associated with greater anxiety and depressive symptoms (Giuntella et al., 2021; Puccinelli et al., 2021). Thus, it is possible that PA and QOL were impacted by the pandemic. Future analyses could seek to understand the impact of pandemic-related factors, including analyzing continuous Fitbit data to provide deeper context for these results.

A few other methodological limitations should be considered. Although the one-arm, pre-post design was appropriate given the focus on exploring feasibility, acceptability, and satisfaction, the lack of a comparison arm or randomization limits evidence of causality. Further, the measures of strength and flexibility were self-report and only captured frequency, rather than duration and/or intensity. The use of a multicomponent intervention with the current study design limits our ability to quantitatively determine which intervention components were most effective and for whom, and to isolate, describe, and evaluate the impact of specific features (i.e., “active ingredients”) on PA and QOL outcomes. Future research should consider alternative experimental approaches, such as MOST, that enable the evaluation of multiple specific intervention components on study outcomes (Collins et al., 2007). For instance, some participants described that joining this study was among their first self-care experiences since their diagnosis, a rich qualitative insight suggestive of improvements in QOL. However, within the current study design, we are limited in determining the relative contributions of increasing PA, engaging with the Fitbit Community, and/or peer mentoring on social support and improvements in QOL. A more nuanced understanding of which intervention components are most potent, which are ineffective, and which are potentially reducing the overall potency of the intervention would support the development of more efficient and effective interventions (Collins et al., 2007).

Characteristics of study participants and how they were recruited may also impact generalizability and impact of the findings. While social media recruitment was cost and time-efficient, participants in this study were likely more technologically savvy and more motivated to change their PA behavior than the general younger breast cancer survivor population. Additionally, while we expected that online recruitment would yield a diverse sample, based on the racial and ethnic diversity of social media users overall (Pew Research Center, 2021), participants were mostly white (88%) and non-Hispanic (85%), and all five peer mentors were white. There are large racial and ethnic disparities in cancer incidence rates, tumor types, and outcomes among young breast cancer survivors. African American and Hispanic women are more likely to be diagnosed at a younger age, with more aggressive forms of breast cancer (i.e., triple negative disease), and are more likely to die from breast cancer compared to other racial and ethnic groups (Carey et al., 2006; DeSantis et al., 2019; Shoemaker et al., 2018). Recruiting participants and peer mentors from diverse racial and ethnic backgrounds will enhance the generalizability of the findings and relevance to the broader young breast cancer survivor community. Since the impact of PA interventions on QOL may vary by ethnicity (Dieli-Conwright et al., 2021), recruitment of diverse study participants is critical to ensure interventions are relevant to the target populations. In addition to posting on the social media pages of groups that serve younger survivors, recruitment for future studies should specifically engage organizations that seek to increase racial and ethnic representation in clinical research, such as For the Breast of Us. Finally, our sample comprised a relatively wide age range (18–54 years old). Younger versus older women within our younger survivor sample may have had different perceptions of intervention components or experienced different impacts in PA and/or QOL outcomes. There may also be differences in intervention experiences and/or outcomes based on time post-treatment, but this study did not collect end of treatment date. Future studies with larger samples and greater statistical power should stratify analyses by different age groups and time since end of treatment to tease out differences, since younger breast cancer survivors are not a homogenous group.

## Strengths

A major strength was the use of CPBR. The community-academic partnership team worked closely to develop research questions, identify meaningful outcomes, and determine how the evidence-based exercise program could be adapted to meet the needs of younger survivors. The strong partnership enhanced the relevance and accessibility of the intervention to the target population. Another key strength was the use of multiple methods and types of data to address the study aims. This approach helped overcome limitations of using any one method and yielded a rich dataset for exploring acceptability of the intervention and benefits to QOL.

## Conclusion

This study showed that a fully remote, peer-to-peer PA intervention is feasible to conduct and acceptable to younger breast cancer survivors. Participants achieved both qualitative and quantitative improvements, some of which reached clinically meaningful thresholds, across multiple QOL domains that are highly impacted in younger cancer survivors (Ruddy et al., 2013). Refinements to the intervention and its delivery should be further assessed in future, more robust studies, toward the ultimate goal of scaling up the intervention for broader dissemination to younger cancer survivors. In closing, this study provides preliminary evidence that peer support and technology can be coupled to provide a supportive, accessible intervention to improve the health and QOL of the rising number of younger women diagnosed with breast cancer, an understudied and overburdened population in need of intervention.

## Electronic supplementary material

Supplementary Material 1
