# Supplementary material for: A remotely delivered, peer-led intervention to improve physical activity and quality of life in younger breast cancer survivors
Source: J Behav Med. 2022 Dec 7;46(4):578–93. doi: 10.1007/s10865-022-00381-8 (PMC9735111; doi:10.1007/s10865-022-00381-8)
Supplement: Supplementary file 1 — Supplementary Material 1 [file 10865_2022_381_MOESM1_ESM.docx]

**Supplementary Material 1**

1. **Pink Body Spirit – 3 Month Qualitative Interview Guide**

#### I. Peer Mentor

1. Tell me what it was like working with your peer mentor, a fellow young breast cancer survivor
   - *What did you like most about working with your mentor?*
   - *What would have made working with your peer mentor more helpful?*
   - *What did you think about working together without seeing each other in person (over video chat)?* How did this method of communication impact how you worked together?

#### II. Fitbit

1. Tell me about your experience using the Fitbit tracker
2. Tell me about your experience using the Fitbit app
3. Tell me about your experience with the Fitbit Community. What would have made the Fitbit community more helpful?
4. What other tools did your peer mentor suggest to help you increase your exercise?
   - *What did you like or not like about XXX? What would have made XXX more useful?*

#### III. Health benefits

1. What benefits have you experienced from participating in this program?
   - *Mental health benefits, physical health benefits, other benefits?*

IV. Program overall

1. How do you feel about the program overall?
2. What suggestions do you have for improving the program?
3. Were there things you were hoping to have or do as part of this program that you didn’t get?
4. What will it be like for you to maintain your exercise now that the sessions with your peer mentor are over?

#### V. Summary

1. Here are some of the key points that I heard from you today:
   - Did I get this right? Was there anything I missed? Is there anything else that I didn’t ask about, but should have?
2. **Qualitative interview rapid analysis approach**
3. To begin, a neutral domain name was selected that corresponded with each section of the semi-structured interview guide. The domain names were working with a peer mentor; remote, technology-based intervention delivery format; Fitbit tracker and app; Fitbit community; perceived benefits of participation; and perspectives on maintaining exercise behavior change during the 12-week post-intervention follow-up period.
4. An Excel matrix organized as participant (rows) by interview domain (columns) (participant x domain) was created. Key points from the project manager’s post-interview field notes were verified against the transcripts, and line numbers of key points in the transcripts were noted. The project manager then transferred verified key points for each domain into the participant x domain matrix, which provided a way to view key similarities and differences for each domain across participants.
5. Key points for each domain were validated with subject matter expert and co-PI SJH. The key point validation process was designed to enhance the consistency and validity of interpretations of data. Interviews were split into three cohorts in chronological order of when they were conducted (the first 5 interviews, the next 10 interviews, and the final 14 interviews) and three separate key point validation sessions, one session for each cohort, were conducted. The first session consisted of the smallest sample of interviews (n = 5) to allow the validation team to explore key points in more detail. The cohort sizes were enlarged for the second and third validation sessions to broaden the possible range of responses available during the validation process. Key point validation sessions aimed to build team consensus around key points that had been identified by the project manager and offered the opportunity to enhance or elaborate on interpretations of key points. If any points needed clarification, line numbers of key points in transcripts were referenced and key point statements were revised, as necessary. During each session, the focus was on validating key points from the new interviews that had been added to the matrix since the last key point validation session.
6. After each key point validation session, the project manager created a list of main themes for each domain. Once all three key point validation sessions had occurred and key points across all interviews were validated, lists of themes from each validation session were triangulated and descriptive summaries were developed to describe main themes for each domain.
